# Emergence of a Snake-Like Structure in Mobile Distributed Agents: An Exploratory Agent-Based Modeling Approach

**DOI:** 10.1155/2014/140309

**Published:** 2014-02-20

**Authors:** Muaz A. Niazi

**Affiliations:** ^1^Bahria University, Islamabad 44000, Pakistan; ^2^COSIPRA Lab, Computing Science & Mathematics, School of Natural Sciences, University of Stirling, Stirling FK9 4LA, UK

## Abstract

The body structure of snakes is composed of numerous natural components thereby making it resilient, flexible, adaptive, and dynamic. In contrast, current computer animations as well as physical implementations of snake-like autonomous structures are typically designed to use either a single or a relatively smaller number of components. As a result, not only these artificial structures are constrained by the dimensions of the constituent components but often also require relatively more computationally intensive algorithms to model and animate. Still, these animations often lack life-like resilience and adaptation. This paper presents a solution to the problem of modeling snake-like structures by proposing an agent-based, self-organizing algorithm resulting in an emergent and surprisingly resilient dynamic structure involving a minimal of interagent communication. Extensive simulation experiments demonstrate the effectiveness as well as resilience of the proposed approach. The ideas originating from the proposed algorithm can not only be used for developing self-organizing animations but can also have practical applications such as in the form of complex, autonomous, evolvable robots with self-organizing, mobile components with minimal individual computational capabilities. The work also demonstrates the utility of exploratory agent-based modeling (EABM) in the engineering of artificial life-like complex adaptive systems.

## 1. Introduction

Legless and mostly shy snakes still have a lot to offer in terms of knowledge as well as an impact on our everyday lives as well as the economy. Though not particularly liked by most humans in the civilized world [1], snakes play a key role in our planet's living ecosystem [2]; they control global rodent populations. Since rodents are well-known vectors of numerous diseases [3], it implies that snakes indirectly and invisibly also assist us in limiting the spread of numerous diseases, which in turn is linked with a country's healthcare and economy. Additionally, snakes are also extraordinary examples of resilience—to the extent that some species have even been labeled “biotic invaders” [4].

Arguably, one of the best and most prevalent ways of learning from nature has been to mimic it—static illustrations from the cave art could quite possibly be considered as one of the earliest forms of recorded expression of human creativity in the form of mimicry of nature. In the current era, computer animations and physical simulations serve a similar purpose. On one hand, we develop models of complex systems to understand how they work and on the other, we can use these models to develop complex autonomous biomimetic or nature-inspired robots and systems to assist in the engineering of our designs. As such, to understand the movement characteristics of snakes and snake-like species, there is a need to be able to develop realistic animations or at least nature-inspired animations. However, making these can actually be a nontrivial problem. While nature is known to exhibit the formation of fairly complex structures based on the interactions of simple parts (or agents), most current paradigms for animation of snakes (either in simulation or in the form of physical robots) are not particularly nature inspired. Examples include [5], which is a robot with wheels. As such, nature presents emergent structures and features (such as the patterns on a Zebra or the structure of complex organs such as the eyes), which are not only complex but also resilient and are made up of numerous replaceable parts. On the other hand, traditional animations of snake-like structures often require mathematically intensive algorithms without actually exhibiting the same level of resilience or complexity as can be easily found in nature. We believe that the missing piece of the puzzle here is that the nature-based approach is differentiated from the typical animation-based approaches by being an agent-oriented approach. In other words, nature enables each particular component to decide and work in tandem with other components using simple interactions, sensing, and messaging.

Since living systems consist of a large number of simple entities acting independently, modeling nature using independent interacting agents is the natural way for developing models of living systems acting as a means of understanding or expanding upon existing natural complex systems [6]. While modeling entities as agents does not inherently require animation, agent-based modeling tools often do offer advanced animation capabilities thereby serving not only as models for understanding the formation of structure (such as reviewed by Méhes and Vicsek in [7]) but also as a means of in silico validation of nature-inspired emergent behaviors and concepts [8].

The particular research problem of interest of this paper is how to develop a nature-inspired, self-organizing agent-based algorithm for the emergent formation of a flexible and resilient snake-like structure, which should be nature-inspired since these approaches though appearing to be “deceptively simple” are often extraordinarily resilient. The key contribution of this paper is an agent-based algorithm for developing simulations of dynamic structures. As a proof of concept, we present extensive simulation experiments which demonstrate the ability of the algorithm to allow a large set of agents, transforming them into a coordinated snake-like structure. We further analyze and study the resilience of the structure and show its application in a 3D environment. We believe that the proposed agent-based algorithm cannot only be used for developing resilient animations of snake-like structures but also in real-world autonomous robots to develop complex, coordinated, and self-organized behavior in agents with loosely coupled structure. In addition, the work can also be considered as a demonstration of life-like artificial complex adaptive system (CAS) requiring minimal intelligence on part of individual agents (or ALife).

### 1.1. Outline

The structure of the rest of the paper is as follows.  Section 2 presents the background.  Section 3 presents the design of algorithms used for the exploratory agent-based model for a self-organized snake-like structure formation. This is followed by a presentation of simulation experiments and a detailed discussion in Section 4. Finally, the paper is concluded with an overview of possible usage and expansions of the proposed algorithm.

## 2. Background

In this section, we first present brief background about the natural structure as well as locomotion in snakes followed by an overview of agent-based modeling for conducting exploratory studies.

### 2.1. Snake Structure and Locomotion

It is a well-known fact that a snake's skeleton is made up of a large number of vertebrae (sometimes up to 300). This large number actually allows for flexibility in the possible variations in the structure of the snake as well as movements even in the absence of well-formed limbs. Additionally, researchers have identified at least 5 basic types of snake locomotion—all made possible primarily due to the flexible body structure. These types include lateral concertina, undulation [9], sidewinding [10], rectilinear [11], and slide-pushing movements [12].

### 2.2. Agents and Exploratory Agent-Based Modeling

A common notion of an agent is “something which acts.” Agents are effective tools for not only modeling complex systems but also as a part of software multiagent systems [13]. Multiagent systems can be developed using various methodologies such as those based on a statechart-based software development process proposed by Fortino et al. in [14]. Not only can agent software be used independently, but they can also be used to develop networked systems as was presented by Aiello et al. in the form of a java-based agent platform for programming sensor networks [15]. Likewise, agents can be online on the web as discussed by Ilie et al. where they examine the flow of online information in an agent-based auction system [16].

Additionally, one way of modeling nature is to use agent based modeling (ABM). ABM is a simulation paradigm which has close ties with actual scientific experiments thus making it a valuable technique for the evaluation of different paradigms or concepts [6]. Most agent-based models start out as exploratory in nature. Some examples of exploratory agent-based modeling studies include work by Palmer et al. for modeling artificial economic life [17], Becu et al. for modeling catchment water management [18], work by Holland [19], by Premo for ethnoarchaeology [20] to work using ABM for modeling AIDS spread [21]. Other examples of exploratory agent-based modeling include a simulation of how research can be considered as an emergent phenomenon as presented earlier in [22].

A unified framework for modeling complex systems using agents and networks of agents has been proposed in the form of a Cognitive Agent-Based Computing (CABC) framework by Niazi and Hussain in [23, 24]. The CABC framework for the modeling and simulation of CAS is structured in the form of 4 different modeling levels - primarily for ease of usage by researchers from different disciplines of science and humanities. These modeling levels are briefly described as follows: The first modeling level of the framework involves the use of complex networks to model, visualize, simulate and analyze any CAS. Exploratory agent-based modeling (EABM) is the second level of this framework. The EABM modeling paradigm allows researchers to experiment and develop proof-of-concept models of CAS with the goal of performing experimentation for improving understanding about a particular real-world complex system. The third level of the framework is DescRiptivE Agent-based Modeling (DREAM). DREAM allows researchers to develop semi-formal, formal or pseudo-code-based specifications coupled with complex network representations of agent-based models allowing models to be better described for communication across disciplines without requiring the same usage of terminology. The fourth modeling level of the framework is the validated agent-based modeling level which involves the creation of a Virtual Overlay MultiAgent System (VOMAS) for ensuring the validity of the simulation model by checking its conformance with the real world.

## 3. Algorithm Design

In this section, we present the design of the two sections of the agent-based algorithm for the autonomous, self-organized formation of the emergent snake-like structure.

### 3.1. Setting Up

The first part of the agent-based paradigm is presented in Algorithm 1. This is primarily required for setting up the environment. It first starts out by clearing any remnants of previous simulations in the agent world. Thus, if any agents were already there or any variables have previously been initialized, they are all cleared. Subsequently, the agent breed “Parts” is used to create a number of agents equal to *n*. It is important to note here that *n* is not only a variable but is also configurable in the model. Next, each of these agents is given a uniform shape (circle) and color (green) for easy identification from other agents.

Each of these agents has an internal variable which identifies whether the agent is a leader or not. After the agents are all placed on random coordinates, they are also all initialized with no parent node and also with no instruction for being followed by other agents.

Now, we can next note here that one of the agents is randomly selected for the purpose of being seeded as the leader. This particular agent has not only a different color for identification but also has the two internal variables of “leader?” as well as “followme?” set to true. Here, we would like to note here that finding a leader in a distributed system is a problem which is very common and has been solved numerous times by using a class of algorithms called the “Election” algorithms [25]. Here, it is also interesting to notice the flexibility and strength of the Logo programming language for executing these agent-based commands which allows for a, more or less, direct translation of the same. In a regular programming language, there would have been a requirement of writing an iterative loop to perform the same tasks going from one agent to the next.

### 3.2. Algorithm for Iterative Agent Function

In the first part of the agent-based paradigm, we have noted an initialization of the structure and agents. In this section, we present the part of the algorithm which is actually called repeatedly as in Algorithm 2. In other words, this algorithm is called in each time step and represents one instance of execution primarily due to the agent-based programming paradigm used here. Here we also note that the algorithm primarily consists of two different sections of commands. The first set of independent commands is for the leader node. The second set of commands is for the rest of the agents. For the leader, the role is quite simple; the agent is essentially requested to move in a random manner. However, for the other agents, there are several different possibilities as follows.

1. Firstly, if the agent is not attached to the snake-like structure, then it will simply do two things.
  - It will move in a random manner.
  - Subsequently, it will check its radius to see if it is near an agent which is the tail end of the snake. This is essentially identified by a “true” value for the Boolean variable “Followme?”. Here, we would like to note that this can be conducted by using messaging between agents. So, each agent acting as a tail can broadcast this information periodically in its communication radius. So, if an agent is indeed in the vicinity of the tail agent, it will itself become the tail node and inform the previous tail agent about this state transition by means of a message.
2. For all other agents, which are attached to the structure by following the nodes, the algorithm requires taking a few random turns and then moving along in a direction 180° to the leader. This is then followed by an adjustment to the heading once again with respect to the parent. Its final movement will be towards the parent but only to half the distance it had previously from the parent. The reason for doing this is to allow for a suitable distance between these nodes. In real-world implementations, this distance could be measured by means of localization algorithms or else by using a global positioning system.

## 4. Results and Discussion

In this section, we describe the outcome of executing the algorithm in different conditions.

### 4.1. Overview of the Environment

**Figure 1:**
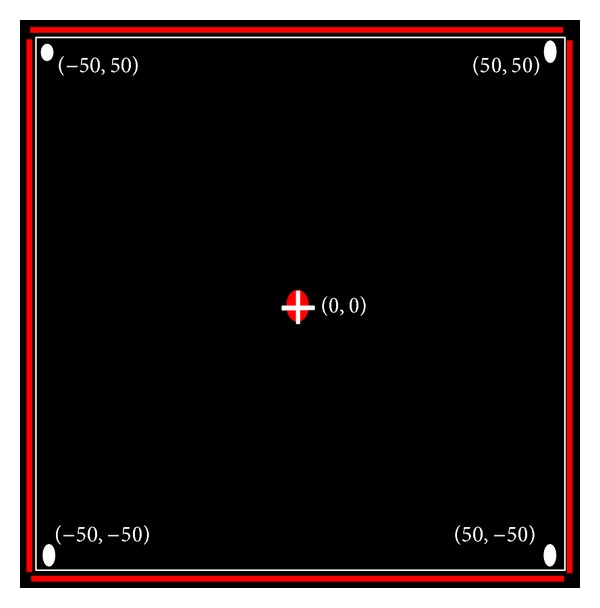
Structure of the world.

The animation environment used for this paper is NetLogo [26]. NetLogo is an ABM tool and is in extensive use by multidisciplinary researchers in domains as diverse as the social sciences to biological and computer sciences. The Logo programming language is based on a graphical “world” and agents living in that environment are termed as “Turtles.” In Figure 1, we present a screen shot depicting the turtle's world. Here, we can note that the world here is based on a 2D Cartesian coordinate system with an origin at the center of the world. This, however, is not an essential condition and can be modified as per requirements. The sides of the world are colored red to demonstrate the rectangular nature. If the sides were open, the structure of the world could essentially be a cylinder (vertical or horizontal), based on the particular sides which were closed or even toroidal in case the top as well as the bottom were left open.

### 4.2. Experiments Set-I: Snake Structure Formation

Here we first discuss the results of basic simulation experiments for the distributed snake-formation algorithms. The environment is shown in detail in Figure 1.

#### 4.2.1. Initialization

**Figure 2:**
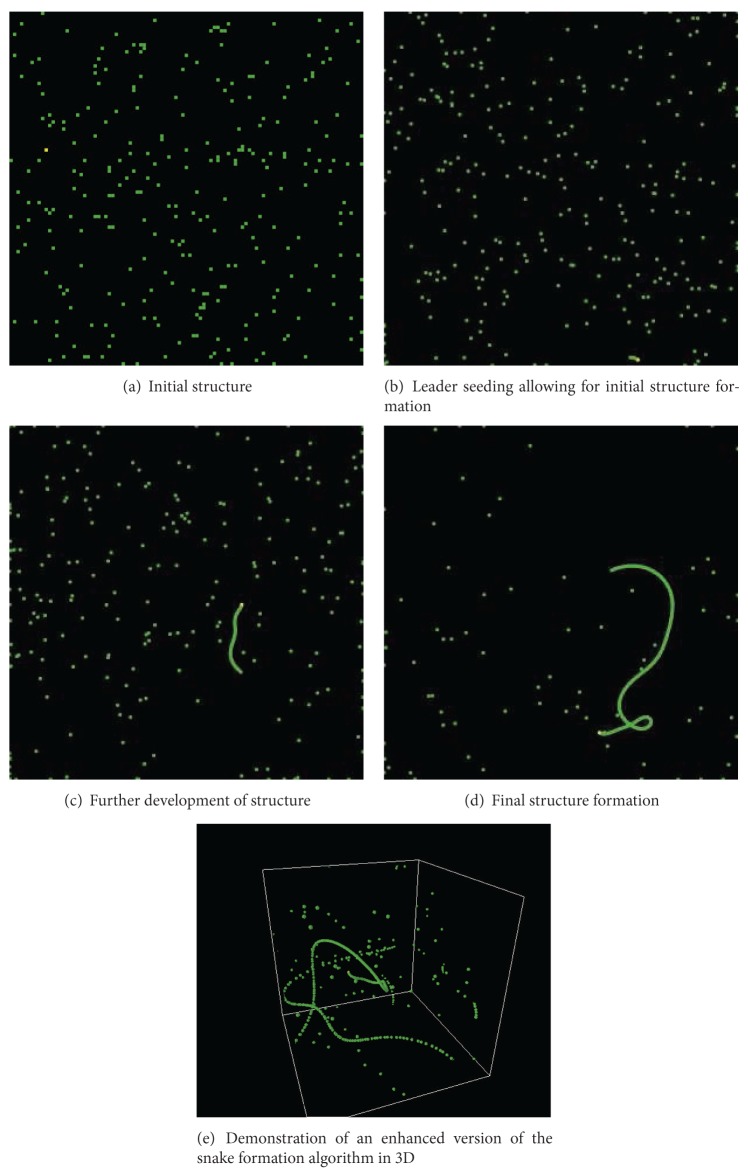
Detailed examination of self-organization.

Initially, the world starts out with a set of agents at random locations. At the start of the simulation, the agents start moving about randomly in the form of a random walk as can be noted in Figure 2(a).

#### 4.2.2. Leader Seeding

The next step in the algorithm is leader seeding. In leader seeding, a random leader is elected. We would like to note here that in case the algorithms were adapted for implementation in autonomous robots, possible ways of leader selection could include the use of election algorithms such as those proposed in [27, 28] or those specifically designed for robot networks [29]. To clearly identify the seeded leader, we give a yellow color to the agent as shown in Figure 2(b).

#### 4.2.3. Structure Formation

The next step in the emergent structure formation is the initialization of the snake-like structure as can be observed in Figure 2(c). It is obvious here that the structure is quite flexible and is also dynamic.

#### 4.2.4. Final Flexible Emergent Structure

Subsequently, this is followed by the final flexible snake-like structure formation as can be noted in Figure 2(d). The beauty about this structure formation is that it is based on the actions and choices of numerous agents without any particular requirement for a specific controlling algorithm governing the system. This entire snake-like structure also keeps on moving around the world—each agent independently responsible for maintaining the structure; in other words, no single agent is important in this emergent large-scale engineered dynamic structure.

#### 4.2.5. Portability of the Algorithm to 3D

To further demonstrate the effectiveness of the algorithm, we demonstrate how the algorithm can easily be ported to 3D in NetLogo 3D. Here, the simulation is being demonstrated using a world with no boundaries so essentially agents can move from one wall to the other. It is interesting to note that the structure is still able to form and function properly maintaining its shape without any problem as can be observed in Figure 2(e).

### 4.3. Experiment Set III: Resilience of the Emergent Structure

In the previous section, we have examined and discussed the large-scale emergent dynamic snake-like structure made from numerous agents. Fault tolerance is an important feature of large-scale distributed systems [30]. The basic idea here is to demonstrate resilience and evaluate fault-tolerance of the dynamic system. This is performed by removing a key component in the dynamic structure—the leader node. The results of this set of experiments are presented next in different stages for the sake of clarity.

#### 4.3.1. Leader Destruction

**Figure 3:**
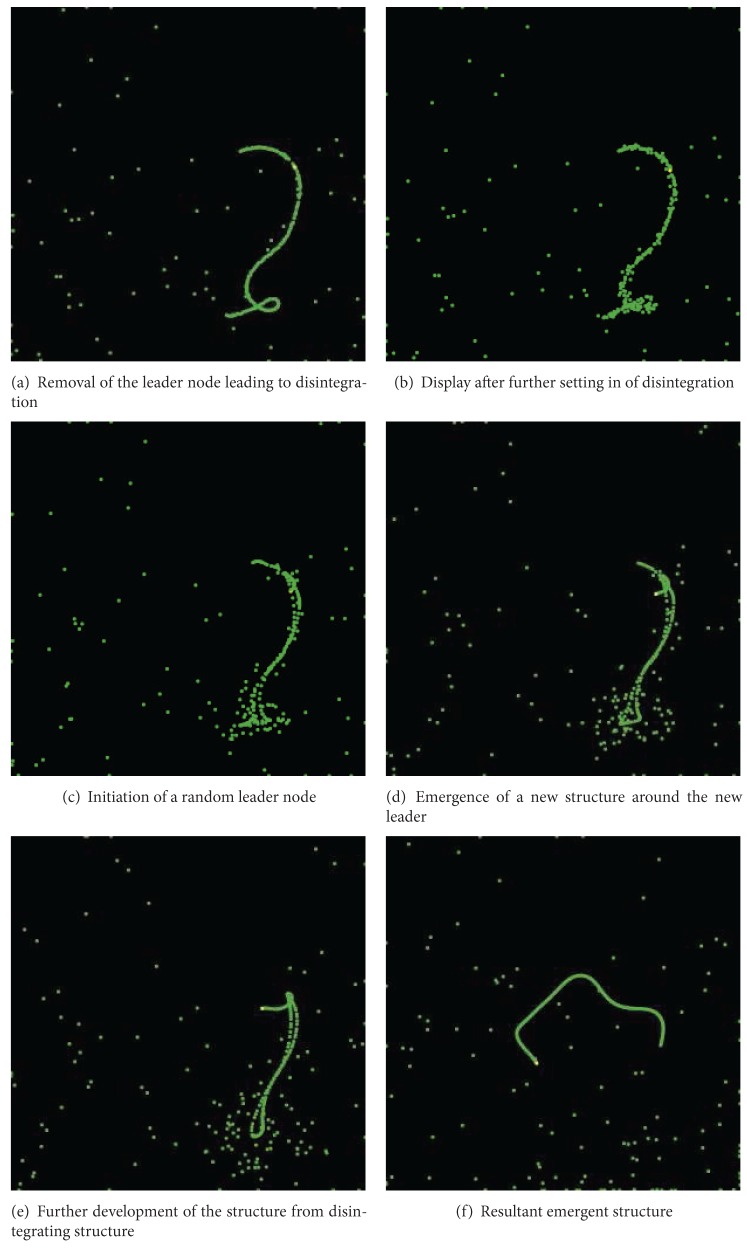
Resilience.

The first step towards measuring resilience in the system is achieved by destroying the leader. We perform this by killing the leader agent as can be observed in Figure 3(a).

#### 4.3.2. Structure Disintegration

As soon as the leader is gone, we note that the entire structure starts disintegrating as can be seen in Figure 3(b). Here, we note how all agents revert back to random walk.

#### 4.3.3. Leader Reseeding

To evaluate the resilience of the structure, next we reseed the system with a leader by executing an election algorithm. We can note here the effects of introduction of a new leader. So, initially while most agents are still doing a random walk, the leader node starts to gather a following. The idea is to further examine how the structure now behaves and if the system recovers within a reasonable amount of time. We can see the effect of this in Figure 3(c).

#### 4.3.4. Reemergence of Structure

Next, we see the reemergence of structure from the ashes of the previous structure as can be noted in Figure 3(d) even while the previous structure is still disintegrating.

#### 4.3.5. Further Development of Emergent Structure

In the next stage, we see further development of the emergent structure in Figure 3(e). Here the structure is already partially formed now, and we note how the agents, which were part of the previous structure, are gradually starting to join their peers in the new structure.

#### 4.3.6. Resultant Emergent Structure

Finally in Figure 3(f), we see the resultant structure which is a flexible copy of the original. The structure is thus clearly resilient and is demonstrated to only require minimal interaction of the simulation agents except for seeding the leader agent/node.

#### 4.3.7. Quantitative Evaluation

**Figure 4:**
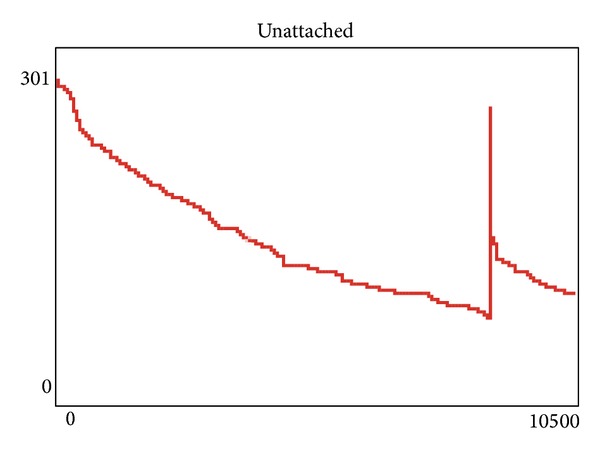
Effect of resilience in the face of losing the leader.

While we have demonstrated an in silico examination of the paradigm, a question can arise as to if we can quantitatively measure the behavior to identify the changes in the system due to destruction of the leader and the subsequent reseeding similar to approaches presented earlier by Niazi and Hussain in [31, 32]. To further evaluate the effects of this, we start by monitoring the unattached nodes versus attached nodes as can be observed in Figure 4. Here, we follow a similar paradigm to these previous papers by doing a summation of the unattached nodes. Now, we can note how the unattached nodes go down over time as the initial structure is formed. However, after a significant amount of time, the initial structure is stable and can be considered as completely formed—something which can now also be noted quantitatively via the plot. Subsequently, however, we see a spike in the plot; this spike shows the effect of the removal or destruction of the leader node.

Subsequent to the removal of the leader agent, we can note here that the number of unattached nodes rises steeply but only for a very brief amount of simulation time. The situation however changes with the election of the new leader agent. This is the reason that the number of unattached nodes now immediately goes significantly down. We also note that even when the spike was high, it never really visibly reaches the same amount of unattached nodes as in the past demonstrating not only resilience but also a “spring” nature, forcing the nodes to quickly regain their previous state of being part of the snake structure. This is due to the resilient nature of the algorithm which is able to quickly reduce the number of unattached nodes. Also, we note here that the system takes a much smaller time to get back to the snake-like dynamic structure as compared with the initial time taken to develop the structure in the first instance. This resilience can perhaps be considered as a proof-of-concept of the effectiveness of the self-organized agent-based approaches, in general and the proposed algorithm, in particular, in the formation of dynamic resilient structures.

### 4.4. Related Work

While, to the best of our knowledge, there is no exact match for a similar agent-based work on snake-like structures, we present here some somewhat related work on snake and worm-like structures. Miller has modeled legless figures such as snakes and worms using systems based on mass-spring concepts [33]. Miller also discusses the possible uses of snake robots in search and rescue in [34]. Steigenberger and Behn have modeled terrestrial locomotion systems based on worms in a straight line using mass point chains along with ground interaction with the help of spike structures making unidirectional motion [35]. Tu presents a paradigm based on artificial life for graphics-based simulation of artificial animals [36]. They use “deformable dynamic contour in the x-y image plane,” essentially physics-based techniques originally proposed by Kass et al. in [37]. Gao et al. explore the influence of a snake on attention and perspective taking using a randomly moving disc as the agent's “head” [38]. Their key results include the revelation that biological agents may be perceived based on rather simple messages resulting in effects on their cognition and perception. Iglesias and Luengo present work assisting the design of behavior which may be helpful for developing closer-to-life animations of virtual agents with a focus on modeling humans [39]. Wong and Datta propose a set of techniques for real-time and realistic rendering as well as animation of small plants [40]. Seol and Noh use a deformation-based approach to develop locomotive animation of a snake [41]. Recent work in the field of biomimetic robots has demonstrated the use of modular molds to construct snake-like robots [42].

## 5. Conclusions

In this paper, we have presented an agent-based algorithm for a resilient snake-like structure formation. It is important to note that while different algorithms are available for developing snake-like structures, the presented algorithm is self-organizing, emergent and is particularly based on the actions of individual agents. In other words, the algorithm is not dependent on a global control for the emerging structure. The structure is therefore completely flexible, self-organizing, and self-adapting.

It is clear that by mimicking the complex adaptive nature of life and associated structures, the presented algorithm results in a system which can be considered as an example of an artificial CAS. Not only is the structure self-organizing as well as emergent in nature, but it also allows for a very interesting resilience even in the absence of the key node required for the formation of the dynamic structure (i.e., the leader node). Thus, even with a removal of this node, it is quite easy for the entire structure to reform within a minimal amount of time. Simulation results demonstrate the effectiveness as well as emergent complexity from simple interactions of the adapting agents in a nature-inspired manner. The results from the set of conducted experiments successfully demonstrate the formation of snake-like structure as well as the resilient nature of the generated structure.

### 5.1. Limitations and Future Work

The proposed algorithm essentially outlines a basic set of ideas useful for developing snake-like autonomous self-adaptive shapes in simulations as well as robotics. Here, we highlight the limitations of the presented work including those pertaining to scope besides presenting some ideas on how the work can be further revised and expanded in the future.

First of all, currently we have not examined the effects of multiple snake-like structure formation in the same simulation. Secondly, we have also not examined how to develop a formal specification model of the system. Furthermore, the effects of obstacles and radio blackout zones in the path of the simulated snake have also not been studied. These could all be possible extensions of our work.

Additionally, while we have conducted simulation experiments, the agent-based algorithm can also be used in the development of real-world decoupled autonomous robots exhibiting complex, coordinated, and self-organized behavior. In addition, the work can also be considered as a demonstration of life-like but artificial CAS requiring minimal intelligence on part of individual agents. Furthermore, in nature ants, termites and other insects are able to develop complex structures using chemical signals, which can also be either simulated or tested using actual robots. The proposed algorithm can be further expanded to develop numerous other types of self-organizing and self-healing dynamic complex structures in autonomous robotic systems for performing complex tasks using a minimal of computational power as well as messaging.

## Figures and Tables

**Algorithm 1:**
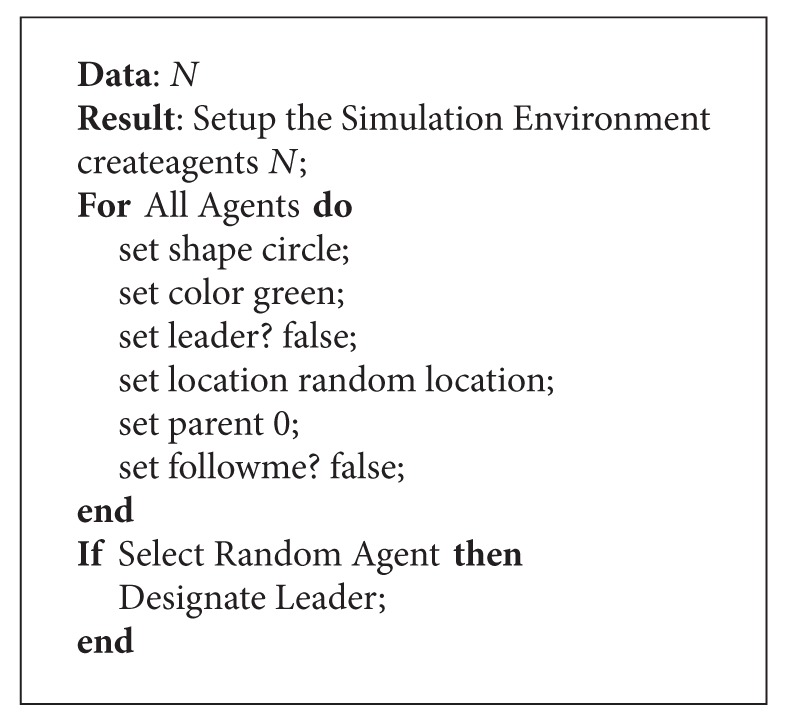
Setting up the simulation environment.

**Algorithm 2:**
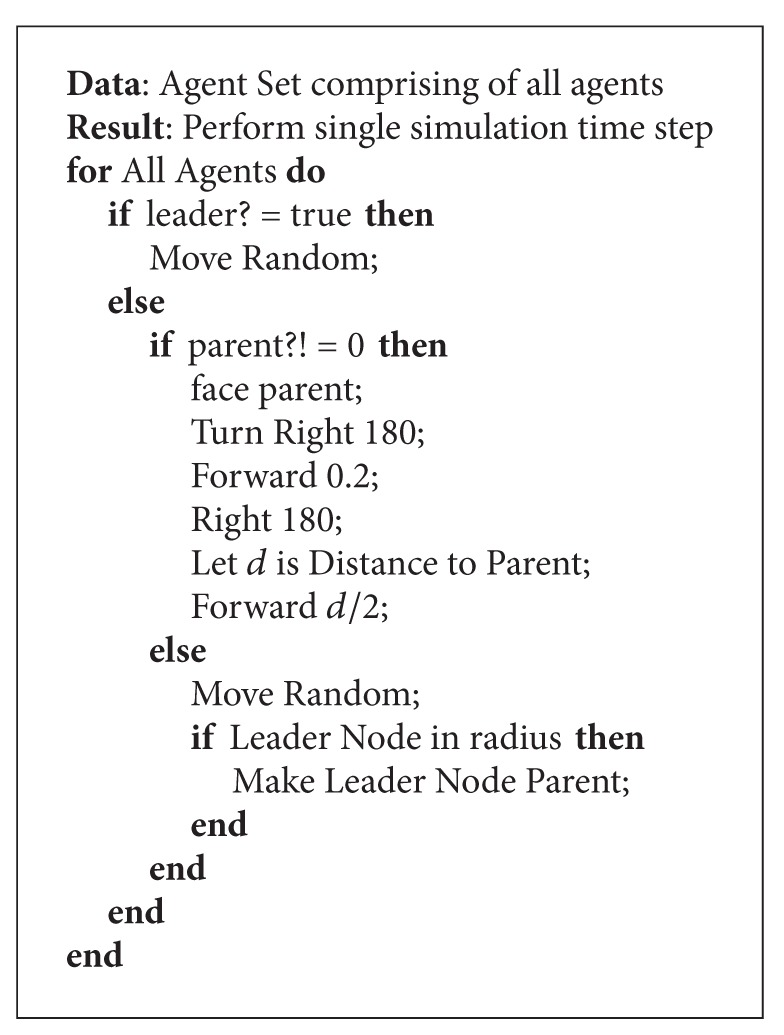
Main animation algorithm.
